# Motor developmental delay in preschoolers with autism spectrum disorders in China and its association with core symptoms and maternal risk factors: a multi-center survey

**DOI:** 10.1186/s13034-025-00858-9

**Published:** 2025-03-05

**Authors:** Dan Long, Ting Yang, Jie Chen, Jie Zhang, Ying Dai, Li Chen, Feiyong Jia, Lijie Wu, Yan Hao, Ling Li, Xiaoyan Ke, Mingji Yi, Qi Hong, Jinjin Chen, Shuanfeng Fang, Yichao Wang, Qi Wang, Chunhua Jin, Tingyu Li

**Affiliations:** 1 Children Nutrition Research Center, Chongqing Key Laboratory of Child Neurodevelopmental and Cognitive Disorders, Ministry of Education Key Laboratory of Child Development and Disorders, Children’s Hospital of Chongqing Medical University, National Clinical Research Center for Child Health and Disorders, Chongqing, 400014 China; 2 Children Health Care Center, Xi’an Children’s Hospital, Xi’an, 710043 China; 3 Department of Developmental and Behavioral Pediatrics, The First Hospital of Jilin University, Changchun, 130021 China; 4 Research Center for Child Development and Behavior, Harbin Medical University, Harbin, 150081 China; 5 Department of Pediatrics, Tongi Hospital of Tongji Medical College, Huazhong University of Science and Technology, Wuhan, 430030 China; 6 Department of Children Rehabilitation, Hainan Women and Children’s Medical Center, Haikou, 570100 China; 7 The Affiliated Brain Hospital of Nanjing Medical University, Nanjing, 210005 China; 8 Department of Child Health Care, The Affiliated Hospital of Qingdao University, Qingdao, 266555 China; 9 Maternal and Child Health Hospital of Baoan, Shenzhen, Shenzhen, 518101 China; 10 Department of Child Health Care, Shanghai Children’s Hospital, Shanghai, 200062 China; 11 Department of Child Health Care, Children’s Hospital Affiliated of Zhengzhou University, Zhengzhou, 450018 China; 12 National Health Commission Key Laboratory of Birth Defect for Research and Prevention, Hunan Maternal and Child Health Hospital, Changsha, 410008 China; 13 Deyang Maternal and Child Health Hospital, Deyang, 618000 China; 14 Department of Children Health Care, Children’s Hospital Affiliated to Capital Institute of Pediatrics, Beijing, 100020 China; 15 Children’s Hospital of Chongqing Medical University, Chongqing, 400014 China

**Keywords:** Autism spectrum disorders, Motor developmental delay, Age, Core symptoms, Maternal risk factors

## Abstract

**Background:**

Motor disturbance, as a related symptom of autism spectrum disorders (ASD), has not received the attention it deserves. We aimed to investigate the different degrees of motor developmental delay and influencing factors in Chinese preschool children with ASD, in order to enhance people’s awareness of motor developmental delay in ASD children.

**Methods:**

We recruited 1,256 ASD children aged 2–6 years from the China Multi-Center Preschool Autism Project (CMPAP). We investigated the overall status of neurodevelopment in preschool children with ASD through the Revised Children Neuropsychological and Behavior Scale (CNBS-R2016) and the Gesell Developmental Scale (GDS). The multivariate ordered logistic regression model was used to analyze the relationship between different degrees of motor developmental delay and demographic, core symptoms of ASD, and maternal risk factors, which were evaluated using the questionnaires, the Childhood Autism Rating Scale (CARS) and the Social Responsiveness Scale-Second Edition (SRS-2).

**Results:**

The proportions of delayed development in various neurodevelopmental domains was significantly imbalanced in preschool children with ASD. The proportions of gross and fine motor developmental delay were as high as 39.6% and 68.4% respectively. ASD children in different age subgroups all exhibited gross and fine motor developmental delay. The CARS and SRS-2 total scores of ASD children with mild, moderate-severe gross or fine motor developmental delay were significantly higher than those with normal motor skills development (*P* < 0.05). ASD children aged ≥ 5 years, or higher CARS and SRS-2 total scores, or gestational age in the 28–36^+ 6^ weeks were more likely to suffer from gross motor developmental delay (OR values were 5.504, 1.083, 1.846 respectively) and fine motor developmental delay (OR values were 2.216, 1.074, 1.011, 1.661 respectively).

**Conclusion:**

Gross and fine motor developmental delay were difficulties that most preschool children with ASD may face, and ASD children with motor developmental delay had greater deficits in social skills. Therefore, it is necessary to continuously monitor the gross and fine motor development progress of children with ASD for facilitating early identification and individualized intervention.

## Introduction

Autism Spectrum Disorders (ASD) are a group of neurodevelopmental disorders characterized by social communication deficits, repetitive stereotypical sensory-motor behaviors, and restricted interests [1]. According to data from the Centers for Disease Control and Prevention in the United States, the prevalence of ASD in 4-year-old children increased from 1.34% in 2010 [2] to 2.15% in 2020; [3] the prevalence of ASD in 5-year-old children in Japan also increased from 0.27% in 2005 [4] to 1.31% in 2019 [5], making it one of the fastest-growing disorders in terms of global prevalence. In 2021, China made the first statistics on the prevalence of ASD in 6-year-old children, which was 1.05% [6]. In recent decades, ASD has emerged as a significant global public health priority, commanding widespread attention from medical experts, policymakers, and the broader society.

Meanwhile, ASD is frequently associated with various comorbidities [6], such as intellectual disability, attention deficit and hyperactive disorder, motor disturbance, etc [1, 7]. Specifically, approximately 35–87% of ASD children exhibit significant motor disturbance [8, 9], while this rate is approximately 4.4% in typically developing children [10]. 

Motor disturbance in people with ASD exhibits high clinical heterogeneity [11], which can manifest as motor developmental delay (MDD), motor coordination disorders, gait abnormalities, and muscle tone abnormalities, etc. MDD mainly refers to a significant delay in milestones of gross or fine motor development in children. The former mainly refers to the displacement and posture maintenance ability of large muscle groups, while the latter mainly refers to the grasping and hand-eye coordination ability of hand muscles. MDD is one of the most prominent features in the early developmental process of ASD, which may has been observed earlier than social communication deficits and language disorders [12, 13], but MDD is not part of the diagnostic criteria for ASD and and has not received the similar attention as social communication deficits. In addition, it has been posited that motor disturbance in children with ASD might be potentially attributed to lack of interest or motivation, difficulty managing social demands, or differences in intellectual ability, and other assorted factors. Therefore, most research on ASD motor are limited to the association with the severity of ASD and intellectual level, but differences in evaluation methods and small sample sizes may led to inconsistent research conclusions [14–18]. Furthermore, there is still a lack of consensus on the relationship between the severity of MDD in ASD children and core symptoms.

On the other hand, the pathogenesis of ASD remains elusive, and it is predominantly postulated that the combined influence of genetic predisposition and environmental factors is at play. Notably, that maternal pre-pregnancy, prenatal and postnatal environmental factors are of cardinal importance in relation to the cerebral development of the fetus [19–23]. After collating data from 37,634 children with ASD and 12,081,416 non-autistic children in 17 studies, scholars further confirmed the relation between maternal risk factors with ASD in offspring, such as parental race being Black and Hispanic, umbilical cord entanglement, premature rupture of membranes, and other factors were not associated with an increased risk of autism; Factors such as offspring being female were associated with a decreased risk of ASD; Factors such as paternal age ≥ 35 years, parental race being White and Asian, gestational hypertension, male offspring, preterm birth, etc. were associated with risk of ASD [24]. In the preliminary research conducted by our research group, based on 57 maternal risk factors, five machine learning algorithms were employed to predict ASD in the offspring. The results indicated that factors such as the mother’s emotional instability and lack of multivitamin supplementation during pregnancy might be associated with an increased risk of autism in offspring [25]. However, the evidence linking the maternal risk factors to the risk of MDD in offspring with ASD is very limited and deserves further investigation.

Therefore, this study originated from the first multi-center investigation on comorbidities and influencing factors of ASD in China. Firstly, we investigated the neurodevelopmental levels of preschool children with ASD. Secondly, we analyzed the association between different degrees of MDD and age, gender, core symptoms of ASD, and maternal risk factors. The aim is to improve people’s awareness and attention to MDD in preschool children with ASD, and to hope to develop individualized intervention measures for MDD in the future.

## Methods

### Study population

The subjects of this study were part of the China Multi-Center Preschool Autism Project (CMPAP, registration number: ChiCTR2000031194) [26]. After obtaining approval from the Medical Ethics Committee of Children’s Hospital of Chongqing Medical University (approval number: (2018) IRB (STUDY) NO.121), an epidemiological investigation of preschool children with ASD was conducted from May 2018 to December 2019. The recruitment domains included 13 cities in five geographical regions of China, including Nanjing and Shanghai in the east; Chongqing, Deyang, and Xi’an in the west; Changsha, Haikou, and Shenzhen in the south; Changchun, Harbin, and Qingdao in the north; and Wuhan and Zhengzhou in the central region. The project included 1,445 ASD children aged 2–6, with an average age of 4.10 ± 1.20; among them, a total of 1,256 preschool children (1,032 boys and 224 girls) with a median age of 3.96 years (interquartile range, 3.18 to 4.89) completed the developmental assessment of gross and fine motor skills and were subsequently included in the final analysis of this study.

The inclusion criteria were as follows: The 2–6 year-old ASD children recruited for this study all come from sub-center outpatient clinics and local special education institutions. They were clinically evaluated by experienced developmental behavioral pediatricians and specialists in child psychology at each sub-center, and diagnosed based on the standard for ASD formulated by Diagnostic and Statistical Manual of Mental Disorders, Fifth Edition (DSM-5) [27]. And all legal guardians of ASD children who agreed to participate in the study signed informed consent form. The exclusion criteria include: (1) Children with brain injuries; (2) Children with severe physical or sensory impairments (blind, deaf); (3) Children with other independent neurodevelopmental disorders and neurological diseases (cerebral palsy, epilepsy); (4) Children who have had acute or chronic infectious diseases in the past 3 months.

The sample size calculation used the formula for population rate estimation based on binomial distribution:

n = (Z^2 * p * (1-p)) / E^2.

Z represents the confidence level (usually taken as 1.96, corresponding to a 95% confidence level), p is the incidence rate. The rate of MDD in children with ASD is set at 35%. E is the allowable error (usually taken as 0.05). The calculated sample size required for this survey is 350 cases.

### Questionnaires and scales

Demographic information (such as name, gender, age, etc.) and maternal risk factors (such as maternal age, emotional state, medical history, vitamin supplementation, gestational age, gravida, birth weight, and cyanosis, etc.) were collected using a questionnaire about children with ASD from their caregivers.

The Revised Children Neuropsychological and Behavior Scale (CNBS-R2016) is the first diagnostic scale for children’s neurodevelopment independently developed in China, which is widely used domestically, and has good reliability and validity [28–30]. The five subscales of CNBS-R2016 are gross motor, fine motor, adaptive behavior, language, and personal-social, and are represented by a developmental quotient (DQ). DQ < 70 points indicates developmental delay (DD), of which 55–69 points indicates mild DD, 35–54 points indicates moderate DD, 20–34 points indicates severe DD, and < 20 points indicates extremely severe DD. A total of 1,021 ASD children were evaluated for motor skills using the CNBS-R2016 in this study.

The Gesell Developmental Scale (GDS) was revised by the Beijing Children’s Health Institute in China and can be used to evaluate children’s development levels [31], showing good consistency with CNBS-R2016 in the evaluation of children’s neurodevelopment [28]. The five subscales of GDS are adaptive behavior, gross motor, fine motor, language, and personal-social behavior, and are represented by a DQ. DQ < 76 points indicates DD, of which 55–75 points indicates mild DD, 40–54 points indicates moderate DD, 25–39 points indicates severe DD, and < 25 points indicates extremely severe DD. A total of 235 ASD children were evaluated for motor skills using the GDS in this study.

The Childhood Autism Rating Scale (CARS) is currently a commonly used diagnostic evaluation tool for ASD both domestically and internationally [31], with good reliability and validity [32], and can be used to evaluate core symptoms of ASD. CARS consists of 15 items, and three subscales: social impairment, negative emotionality, and distorted sensory response [33]. The scale adopts a 7-level scoring method, with a maximum score of 60 points. A total score of 30–36 points, with fewer than 5 items < 3 points, indicate mild-to-moderate; a total score of ≥ 36 points with at least 5 items > 3 points are indicate severe. A total of 813 ASD children were evaluated for core symptoms using the CARS in this study.

The Social Responsiveness Scale-Second Edition (SRS-2) can be used as a screening tool for ASD, with good reliability and validity, and it uses 5 subscales to evaluate the social abilities of ASD [34]: social awareness, social cognition, social communication, social motivation, and autistic mannerisms. The scale consists of 65 items, using a 4-level scoring method. A total score of ≥ 65 points indicate a positive ASD screening. A total of 1,101 ASD children were evaluated for social abilities using the SRS-2 in this study.

### Quality control

Prior to the launch of this study, all researchers were provided with homogenization training, and questionnaires and scales were collected and promptly organized and archived to avoid and reduce data loss. The data was entered via independent double entry and coded for verification to ensure the accuracy and authenticity of the data.

### Statistical analysis

The data were analyzed using SPSS 26.0 software. The Kolmogorov-Smirnov test was used to test the distribution of each dataset for normality. Normally distributed continuous variables were described as means ± standard deviations (M ± SD), while non-normally distributed continuous variables were described as median (p25, p75). Categorical variables were described as n(%). The chi-square test and Kruskal-Wallis rank sum test were used analyze differences between multiple groups. The multivariate ordered logistic regression model was used to explore the impact of demographic, core symptoms of ASD, and maternal risk factors on the level of motor development in ASD. *P* < 0.05 was considered to be statistically significant.

## Results

### Developmental quotient levels in various neurodevelopmental domains of children with ASD

There was a significant imbalance in the proportions of DD among the different neurodevelopmental domains of ASD children (*P* < 0.05). Specifically, the proportion of DD in the gross motor skills was 39.6%, fine motor skills was 68.4%, adaptive behavior skills was 66.2%, language skills was 77.3%, and personal-social skills was 78.3% (Table 1).

**Table 1:** Incidence of developmental delay in various neurodevelopmental domains of 1,256 children with ASD [n(%)]

Items	Gross motor	Fine motor	Adaptive behavior	Language	Personal-social	χ^2^	*P*
**Normal**	758(60.4)	397(31.6)	425(33.8)	285(22.7)	273(21.7)	547.267	0.000
**Developmental delay**	498(39.6)	859(68.4)	831(66.2)	971(77.3)	983(78.3)		
Mild	324(25.8)	398(31.7)	376(29.9)	207(16.5)	335(26.7)		
Moderate	152(12.1)	360(28.7)	345(27.5)	397(31.6)	488(38.9)		
Severe	18(1.4)	88(7.0)	91(7.2)	296(23.6)	133(10.6)		
Extremely severe	4(0.3)	13(1.0)	19(1.5)	71(5.7)	27(2.1)		

ASD, autism spectrum disorders

### The proportion of motor developmental delay in children with ASD in relation to age and gender

Children with ASD were divided into three groups based on their motor skills DQ levels: normal gross or fine motor skills development, mild gross or fine motor developmental delay (GMDD or FMDD), moderate-severe GMDD or FMDD. Delayed development in both gross and fine motor skills was observed in all four age subgroups in this study (Table 2), and the differences in delayed development proportions between subgroups were statistically significant (*P* < 0.05). In the age subgroup < 3 years, 26.8% and 60.2% of children with ASD respectively had delayed development in the gross and fine motor skills. In the age subgroup ≥ 5 years, the proportion of ASD children with moderate-severe GMDD or FMDD was the highest, at 24.7% and 45.0% respectively.

**Table 2:** Comparison of gross/fine motor developmental delay proportions in ASD children in different age and gender subgroups [n(%)]

Items	Gross motor(*n* = 1,256)			Fine motor(*n* = 1,256)		
	Normal range	Mild	Moderate-severe	Normal range	Mild	Moderate-severe
**Total**	758(60.4)	324(25.8)	174(13.9)	397(31.6)	398(31.7)	461(36.7)
**Age**						
<3 years(*n* = 254)	186(73.2)	52(20.5)	16(6.3)	101(39.8)	80(31.5)	73(28.7)
3 ~ 4 years(*n* = 393)	249(63.4)	105(26.7)	39(9.9)	118(30.0)	138(35.1)	137(34.9)
4 ~ 5 years(*n* = 323)	194(60.1)	77(23.8)	52(16.1)	93(28.8)	104(32.2)	126(39.0)
≥ 5 years(*n* = 286)	129(45.1)	90(31.5)	67(23.4)	85(29.7)	76(26.6)	125(43.7)
*H*			55.212			14.335
*P*			0.000			0.001
**Gender**						
Boys(*n* = 1032)	629(60.9)	262(25.4)	141(13.7)	332(32.2)	323(31.3)	377(36.5)
Girls(*n* = 224)	129(57.6)	62(27.7)	33(14.7)	65(29.0)	75(33.5)	84(37.5)
*H*			0.870			0.902
*P*			0.647			0.637

ASD, autism spectrum disorders

The proportion of combined mild, moderate-severe GMDD or FMDD in girls with ASD was higher than in boys with ASD, but the difference was not statistically significant (*P* > 0.05).

### Comparison of core symptom scale scores in children with ASD at different developmental quotient levels of motor skills.

The CARS, SRS-2, and subscales scores of the group with mild, moderate-severe GMDD or FMDD were significantly higher than those of the group with normal motor skills development (*P* < 0.05) (Table 3).

**Table 3:** Comparison of core symptom scale scores in ASD children at different DQ levels of motor development

Items	Gross motor					Fine motor				
	Normal range	Mild	Moderate-severe	H	***P***	Normal range	Mild	Moderate-severe	H	***P***
**CARS(n = 813)**										
Social impairment	22.5(20,26)	24(21.5,27)	26.73 ± 4.76	75.984	0.000	21.44 ± 4.01	24(20,27)	25(22,28.75)	91.816	0.000
Negative emotionality	6(5,7)	6(5,7)	7(6,7.5)	39.584	0.000	6(5,7)	6(5,7)	6(5,7)	17.535	0.000
Distorted sensory response	6(5,7)	6(5,7)	7(6,8)	40.503	0.000	6(4,6)	6(5,7)	6(5,7.75)	19.351	0.000
CARS total score	32(28,36.5)	35(31,38.25)	38.30 ± 7.40	72.602	0.000	31(27,35)	34.38 ± 6.61	36(31,41)	68.432	0.000
**SRS-2 (n = 1101)**										
Social awareness	11(9,13)	12(9,14)	12(10,14)	10.903	0.000	11(8,13)	11(10,13)	12(10,14)	24.408	0.000
Social cognition	18(15,21)	19(15,22)	18(15,21)	8.049	0.018	16.80 ± 4.87	18(15,21)	19(16,22)	34.722	0.000
Social communication	31.5(26,38)	33.18 ± 8.79	34.24 ± 8.46	10.782	0.005	30(23,36)	32(28,39)	34.19 ± 8.28	40.008	0.000
Social motivation	14(11,18)	16(12.5,19)	16(13,19)	20.505	0.000	13(10,16)	15(12,19)	16(13,19)	46.329	0.000
Autistic mannerisms	12(9,16)	14(9,18)	13.93 ± 6.03	11.344	0.003	11.5(8,16)	13.76 ± 6.13	14(10,17.5)	27.031	0.000
SRS-2 total score	87(73,102)	89(79,109)	94.60 ± 21.91	16.066	0.000	82.90 ± 24.35	91.94 ± 23.00	93(81.5,110)	51.572	0.000

ASD, autism spectrum disorders; CARS, Childhood Autism Rating Scale; SRS-2, Social Responsiveness Scale-Second Edition

### Comparison of maternal risk factors in children with ASD at different developmental quotient levels of motor development

Based on the preliminary research by our research group, the top 10 weighted maternal risk factors included in ASD are maternal age, maternal emotional state (emotional stability or instability), medical history (bacterial or virus infections, threatened miscarriage), vitamin supplementation (folic acid or multivitamin), gestational age, gravida, birth weight of offspring and whether cyanosis occurred [25]. 

Compared to the ASD group with normal gross motor skills development, the proportions of mothers not supplementing with folic acid or multivitamin during pregnancy and the gestational age being between 28–36^+ 6^ weeks were higher in the groups with mild, moderate-severe GMDD (*P* < 0.05). There were no statistically significant differences in the proportions of maternal age, emotional stability during pregnancy, infection, threatened miscarriage, gravida, birth weight of offspring and whether cyanosis occurred among the three different groups of gross motor development levels (*P* > 0.05) (Table 4).

**Table 4:** Comparison of maternal risk factors in ASD children at different DQ levels of motor development

Items	Gross motor					Fine motor				
	Normal range	Mild	Moderate-severe	H	***P***	Normal range	Mild	Moderate-severe	H	***P***
**Maternal age**										
20–34 years(*n* = 1045)	635(88.8)	269(86.5)	141(86.0)	1.687	0.430	334(87.9)	326(87.4)	385(88.1)	0.096	0.953
≥35 years(*n* = 145)	80(11.2)	42(13.5)	23(14.0)			46(12.1)	47(12.6)	52(11.9)		
**Emotional stability**										
No(*n* = 408)	238(33.6)	105(35.1)	65(39.6)	2.174	0.337	134(35.7)	118(32.0)	156(36.4)	1.949	0.377
Yes(*n* = 764)	471(66.4)	194(64.9)	99(60.4)			241(64.3)	251(68.0)	272(63.6)		
**Infection**										
No(*n* = 879)	530(75.5)	230(74.4)	119(72.6)	0.639	0.726	289(76.1)	277(74.1)	313(74.3)	0.469	0.791
Yes(*n* = 296)	172(24.5)	79(25.6)	45(27.4)			91(23.9)	97(25.9)	108(25.7)		
**Threatened miscarriage**										
No(*n* = 850)	546(81.9)	206(78.9)	98(77.2)	2.096	0.351	320(81.0)	269(79.1)	2761(81.6)	0.708	0.702
Yes(*n* = 205)	121(18.1)	55(21.1)	29(22.8)			75(19.0)	71(20.9)	59(18.4)		
**Folic acid**										
No(*n* = 221)	115(19.9)	63(25.5)	43(30.1)	8.151	0.017	64(20.5)	67(21.9)	90(25.6)	2.676	0.262
Yes(*n* = 748)	449(80.1)	184(74.5)	100(69.9)			248(79.5)	239(78.1)	261(74.4)		
**Multivitamin**										
No(*n* = 687)	391(57.6)	182(63.0)	114(74.5)	15.531	0.000	192(53.6)	231(64.3)	264(65.3)	13.053	0.001
Yes(*n* = 434)	288(42.4)	107(37.0)	39(25.5)			1766(46.4)	128(35.7)	140(34.7)		
**Gestational age**										
28–36^+6^ weeks(*n* = 135)	68(9.7)	36(11.9)	31(19.3)	11.688	0.003	32(8.6)	41(11.2)	62(14.5)	6.590	0.037
37 ~ 42 weeks(*n* = 1029)	633(90.3)	266(88.1)	130(80.7)			338(91.4)	324(88.8)	367(85.5)		
**Gravida**										
Once(*n* = 763)	473(66.6)	194(63.0)	96(57.5)	5.272	0.072	254 (67.6)	241(64.8)	268(61.3)	3.451	0.178
≥2 times(*n* = 422)	237(33.4)	114(37.0)	71(42.5)			122(32.4)	131(35.2)	169(38.7)		
**Birth weight(kg)**	3.3(3,3.6)	3.3(3,3.6)	3.3(3,3.625)	1.438	0.487	3.4(3,3.61)	3.3(3,3.6)	3.3(3,3.6)	4.501	0.105
**Cyanosis**										
No(*n* = 1009)	609(94.7)	261(93.5)	139(92.1)	1.700	0.427	320(93.6)	322(95.0)	367(93.6)	0.797	0.671
Yes(*n* = 64)	34(5.3)	18(6.5)	12(7.9)			22(6.4)	17(5.0)	25(6.4)		

ASD, autism spectrum disorders; kg, kilogram

Compared to the ASD group with normal fine motor skills development, the proportions of mothers not supplementing with multivitamin during pregnancy and the gestational age being between 28–36^+ 6^ weeks were higher in the groups with mild, moderate-severe FMDD (*P* < 0.05). There were no statistically significant differences in the proportions of maternal age, emotional stability during pregnancy, infection, threatened miscarriage, folic acid supplementation, gravida, birth weight of offspring and whether cyanosis occurred among the three different groups of gross motor development levels (*P* > 0.05).

### Delayed development in both gross and fine motor skills is associated with multiple risk factors in children with ASD

Using different DQ levels of motor development as the dependent variable, the age children with ASD, total CARS score, total SRS-2 score, maternal folic acid or multivitamin supplementation during pregnancy, and gestational age, which show significant differences between the groups, were taken as independent variables. These were included in multivariate ordered logistic regression model to analyze the risk factors influencing MDD in children with ASD. We assigned each independent variable (Table 5).

**Table 5:** Variable names and assignments

Items	Assignment
**Gross motor and fine motor**	0=Normal range, 1 = Mild, 2 = Moderate-severe
**Age**	1=≥5 years, 2=4–5 years, 3=3 ~ 4 years, 4=<3 years
**Folic acid**	1=No, 2=Yes
**Multivitamin**	1=No, 2=Yes
**Gestational age**	1=28 ~ 36^+ 6^ weeks, 2=37 ~ 42 weeks

Children with ASD in the older age subgroup were more likely to suffer from GMDD or FMDD. For example, the likelihood of older children (≥ 5 years) with ASD suffering from GMDD or FMDD were 5.504 times (95% CI: 3.202–9.461; *P* < 0.05) and 2.216 times (95% CI: 1.439–3.411; *P* < 0.05) higher than that of younger children (< 3 years) with ASD, respectively (Table 6).

**Table 6:** Multivariate ordered logistic regression analysis on the influencing factors of motor developmental delay in ASD children

Influencing factors	Gross motor		Fine motor	
	OR (95%CI)	*P*	OR (95%CI)	*P*
Age(reference = < 3 years)				
3 ~ 4 years	1.936 (1.154, 3.247)	0.012	2.074 (1.393, 3.086)	0.000
4 ~ 5 years	3.636 (2.133, 6.200)	0.000	2.473 (1.617, 3.782)	0.000
≥ 5 years	5.504 (3.202, 9.461)	0.000	2.216 (1.439, 3.411)	0.000
**CARS total score**	1.083 (1.055, 1.113)	0.000	1.074 (1.049, 1.099)	0.000
**SRS-2 total score**	1.002 (0.994, 1.010)	0.686	1.011 (1.004, 1.017)	0.002
**Folic acid (reference = Yes)**	1.159 (0.784, 1.713)	0.459	/	/
**Multivitamin (reference = Yes)**	1.250 (0.865, 1.808)	0.235	1.237 (0.920, 1.663)	0.159
**Gestational age (reference=37 ~ 42 weeks)**	1.846 (1.123, 3.034)	0.016	1.661 (1.051, 2.627)	0.030

ASD, autism spectrum disorders; CARS, Childhood Autism Rating Scale; SRS-2, Social Responsiveness Scale-Second Edition; CI, confidence intervals; OR, odds ratio.

The total CARS score of children with ASD was related to their levels of gross and fine motor skills development. The higher the total score of CARS, the greater the likelihood of GMDD or FMDD, with odds ratios of 1.083 (95% CI: 1.055–1.113; *P* < 0.05) and 1.074 (95% CI: 1.049–1.099; *P* < 0.05), respectively.

The total SRS-2 score of children with ASD was related to their levels of fine motor skills development, rather than gross motor skills development. The higher the total score of SRS-2, the greater the likelihood of FMDD, with an odds ratio of 1.011 (95% CI: 1.004–1.017; *P* < 0.05).

The gestational age of ASD children was related to their levels of gross and fine motor skills development. Compared to children with ASD at 37 to 42 weeks of gestation age, children with ASD at 28–36^+ 6^ weeks of gestation age were 1.846 times (95% CI: 1.136 to 3.139; *P* < 0.05) and 1.661 times (95% CI: 1.051 to 2.627; *P* < 0.05) more likely to suffer from GMDD or FMDD, respectively.

## Discussion

Since the first reports of ASD by Kanner and Asperger [35, 36], the clinical heterogeneity of motor disturbance had been continuously described and considered as one of the relevant features of ASD [27]. However, people have always focused on evaluating and intervening in the core symptoms of ASD, largely ignoring the potential mutual influence between motor development and core symptoms [9]. Therefore, this study first investigated the overall situation of DD in five neurodevelopmental domains of children with ASD from a multicenter perspective. It was found that there was a significant imbalance in the proportions of DD among each neurodevelopmental domains, the highest proportions of DD observed in personal-social and language skills, at 78.3% and 77.3% respectively, while the lowest proportion of DD was in gross motor skills, at 39.6%. As a result, caregivers and clinical physicians usually payed more attention to the social communication and language development of ASD children, potentially ignoring the risks associated with MDD. Given that motor disturbance was commonly prevalent in most ASD children in observational research studies but only reported by diagnosing clinicians in around 1% of ASD children in practice [8], increasing awareness is an initial and crucial step toward bridging the research-to-practice gap. Additionally, the proportions of mild, moderate-severe GMDD or FMDD in girls with ASD were slightly higher than those in boys, but there were no statistically significant differences, which was similar to the results of a single-center Chinese study of 219 children with ASD reported by Zhou Bingrui et al. [16], indicating that there were only subtle gender differences in motor development in ASD children. However, Craig et al. [37] found that compared to boys with ASD, girls had significantly higher levels of fine motor skills development, and lower levels of gross motor skills development. It should be noted that the above-mentioned studies had differences in sample size and evaluation tools, and increasing the sample size and unifying evaluation tools may be one of the effective methods for further exploring gender differences in ASD.

Additionally, GMDD and FMDD were observed in all age subgroups included in this study, with over 1/4 and 1/2 of children with ASD exhibiting DD in gross and fine motor skills, respectively. Recent meta-analyses have also shown [38, 39] that significant differences in motor development levels could be observed in children with ASD at 13 months of age, much earlier than the typical age of ASD diagnosis, indicating that motor developmental evaluation has important clinical significance for early detection and referral decision-making in ASD. It is worth noting that this study also found that the likelihood of older children (≥ 5 years) with ASD suffering from GMDD or FMDD were highest, and their motor disturbance became more prominent, which may be related to the inclusion of samples from local special education institutions, whose disease severity is more severe; and the motor development levels of ASD children were always compared to the scales’ normative standards-stricter norms for older children (≥ 5 years) with ASD contributed to the higher observed proportions of GMDD and FMDD. These results reflected the ongoing and increasing difficulties that ASD children face in meeting developmental milestones. In future research, it is necessary to expand the sample source channels and longitudinally follow up the changes in motor development levels of children with ASD in the same group.

On the other hand, there is a growing recognition of the intrinsic connection between motor skills and cognitive, adaptive, and social skills. It is now widely believed that motor skills development plays a crucial role in early social communication, influencing children’s visual and tactile input, as well as their interactions with others and environment [11, 40, 41]. This study showed that ASD children who with mild, moderate-severe GMDD or FMDD exhibited significantly higher symptom scores than those with normal motor skills development. Furthermore, the likelihood of children with ASD suffering from GMDD or FMDD increases with higher total scores on the CARS or SRS-2, indicating a potential mutual influence between motor development levels and core symptoms of ASD, especially social skills. Recent analyses of the SPARK dataset al.so support a similar association between motor and social skills in ASD, with the correlation between social skills deficits and motor disturbance being stronger than that with intellectual ability [42, 43]. Moreover, the early development of gross and fine motor skills in infants with ASD may also have a cascading effect on the future development of social skills [39]. Therefore, understanding the relationship between motor and social skills may guide new clinical evaluations priorities and elucidate mechanisms for intervention targets.

This study also found that the proportions of mothers of ASD children with mild, moderate-severe GMDD or FMDD did not supplement with folic acid or multivitamin during pregnancy were higher than those of ASD children with normal motor skills development. Previous research results also suggested [20] that maternal inadequate nutrient intake can affect fetal brain development. For example, ASD offspring of mothers who did not supplement with folic acid during pregnancy were found to have more severe autism symptoms and DD in gross motor skills compared to offspring of mothers who supplemented with folic acid [44]. However, a meta-analysis of 756,365 children from 10 countries showed that prenatal folic acid supplementation reduced the risk of ASD in offspring by 58%, but had no significant impact on the motor development of the offspring [45]. The effectiveness of maternal folic acid supplementation was often evaluated without considering the impact of genetic factors in these studies [45], as well as the supplementation time and dosage. However, these findings all emphasized the importance of maternal folic acid and multivitamin supplementation during pregnancy for the neurodevelopment of their offspring. Future research can continue to explore the biological mechanisms of vitamins in brain development.

The results of this study are consistent with previous research [23, 46]. We found that the proportion of ASD children with GMDD or FMDD was higher for maternal gestational age of 28–36^+ 6^ weeks compared to children with normal motor development. Furthermore, the multivariate ordered logistic regression model demonstrated that maternal gestational age of 28–36^+ 6^ weeks is a risk factor for ASD offspring with GMDD or FMDD. Although not all premature ASD children would suffer from motor development delay, the focus of caregivers often shifts away from motor developmental milestones after the early developmental period. Therefore, for premature ASD children who have already reached motor developmental milestones, clinical physicians should closely monitor their future neurodevelopmental development, and adding motor disturbance as a specifier would bring needed attention to this issue in the caregiver in order to take early intervention measures.

From the review of literature, current research has not been able to conclusively use motor disturbance as an early marker for diagnosis of autism [47]. However, given that motor disturbance is a common and overlooked feature of autism, some scholars suggested adding a domain-specific ‘specifier’ to the ASD diagnostic criteria [8] to ensure motor difficulties feature more prominently within ASD evaluation early in life, along with greater consideration of how impairment domain may interact with other developmental and clinical domains. Such an addition would signal the need for more targeted clinical attention to motor disturbance and provide a framework for clinicians to better identify and address this issue.

This study has other limitations which need to be addressed. Firstly, the sample for this study was derived from outpatient clinics and special education institutions, which included ASD children who also had global developmental delay or intellectual disability. Furthermore, the influence of comorbidities on motor skills were not further analyzed. Secondly, this study was designed as a cross-sectional study and did not utilize more motor skills evaluation tools, limiting the ability to investigate the specific relationship between motor skills and ASD. Therefore, it is necessary to evaluate co-occurrence symptoms of children with ASD using multiple scales, and implement prospective cohort studies to further explore the essence of the interrelationships between motor disturbance, autistic symptoms, and broader neurodevelopmental disorders.

## Conclusion

In summary, we found that there was a significant imbalance in the proportions of of delayed development of various neurodevelopmental domains among children with ASD. 39.6% and 68.4% of ASD children exhibited GMDD or FMDD, and the likelihood of MDD being greatest in the age subgroup ≥ 5 years, and increasing with higher CARS or SRS-2 total scores, potentially indicating mutual influence with social skills. Furthermore, there was a significant association between maternal gestational weeks of 28–36^+ 6^ and the risk of GMDD or FMDD in ASD children. However, motor disturbance may remain an underutilized area in the diagnosis and intervention of ASD. Therefore, while diagnosing ASD, it is crucial to accurately evaluate the gross and fine motor development levels, and continuously monitor their developmental progresses in motor skills and other domains to achieve early identification and individualized intervention.

## Data Availability

The raw data supporting the conclusions of this article will be made available by the authors, without undue reservation.
